# Immune Reconstitution Inflammatory Syndrome Occurring in a Kidney Transplant Patient with Extrapulmonary Tuberculosis

**DOI:** 10.1155/2017/6290987

**Published:** 2017-03-07

**Authors:** Jose Iglesias, Kandria Jumil Ledesma, Paul J. Couto, Jessie Liu

**Affiliations:** ^1^Department of Nephrology, Jersey Shore University Medical Center, Neptune, NJ 07753, USA; ^2^Rowan University School of Osteopathic Medicine, Stratford, NJ 08084, USA; ^3^American University of Antigua College of Medicine, Coolidge, Antigua and Barbuda; ^4^Ocean Renal Associates, 210 Jack Martin Boulevard, Brick, NJ 08724, USA

## Abstract

Tuberculosis (TB) occurring in solid organ transplantation (SOT) is associated with significant morbidity and mortality usually due to delays in diagnosis, drug toxicity encountered with antimycobacterial therapy, and drug-drug interactions. TB in SOT patients may mimic other infectious and noninfectious posttransplant complications such as posttransplant lymphoproliferative disorder (PTLD) and systemic cytomegalovirus infection. Immune reconstitution inflammatory syndrome (IRIS) is a host response resulting in paradoxical worsening of an infectious disease which occurs after the employment of effective therapy and reversal of an immunosuppressed state. We describe the development of immune reconstitution inflammatory syndrome (IRIS), a unique complication occurring during the treatment of extrapulmonary tuberculosis occurring after transplant which resulted from decreasing immunosuppression in a patient who received Alemtuzumab induction therapy. Although (IRIS) has been originally described in HIV/AIDS patients receiving highly active antiretroviral therapy (HAART), solid organ transplant recipients with diagnosed or occult TB whose immune system may undergo immune reconstitution during their posttransplant course represent a new high risk group.

## 1. Introduction

SOT recipients have a high risk of developing TB during the posttransplant period usually due to reactivation of latent infection [1, 2]. Extrapulmonary and disseminated TB is a common presentation and poses diagnostic challenges and delays in diagnosis [3–5]. Treatment of TB in patients who have received SOT poses many therapeutic dilemmas due to drug interactions and drug toxicity [6, 7]. IRIS is a recognized complication of HIV/AIDS patients with TB undergoing HAART [8–10]. IRIS has recently been described with increasing frequency in solid organ transplant recipients who are undergoing treatment for TB and other opportunistic infections who have necessarily had to have their immunosuppression curtailed [9, 10]. Although IRIS has been increasingly reported in SOT recipients, this entity may still remain unrecognized. Underrecognition of IRIS in these patients may lead to extensive testing, unnecessary changes in therapy, and decreases in immunosuppression. We present an illustrative case of IRIS during treatment of nonpulmonary TB in the setting of Alemtuzumab induction therapy occurring 3 months after renal transplantation.

## 2. Case Report

**Figure 1:**
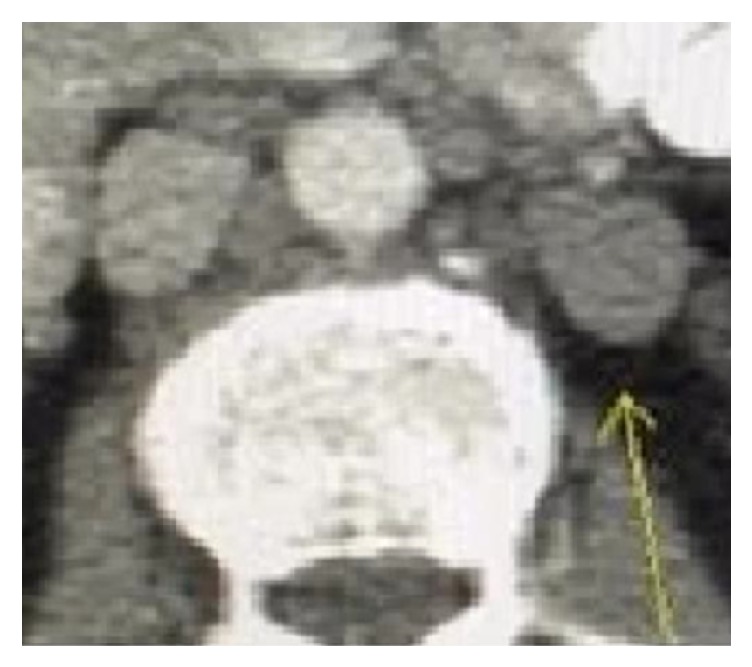
CT scan revealing para-aortic lymphadenopathy.

**Figure 2:**
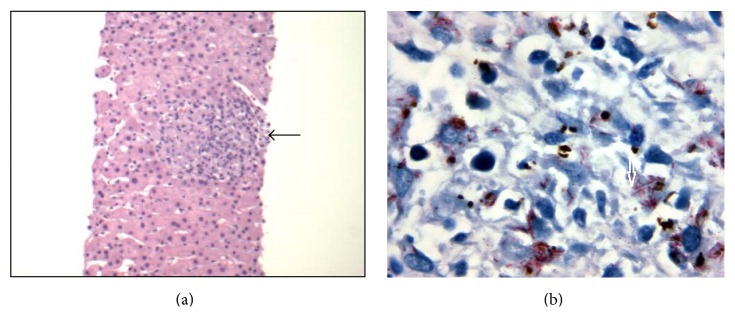
(a) Liver biopsy with Hematoxylin and Eosin stain demonstrating a noncaseating granuloma. (b) Para-aortic lymph node biopsy with Acid fast stain demonstrating the presence of multiple acid fast bacilli.

**Figure 3:**
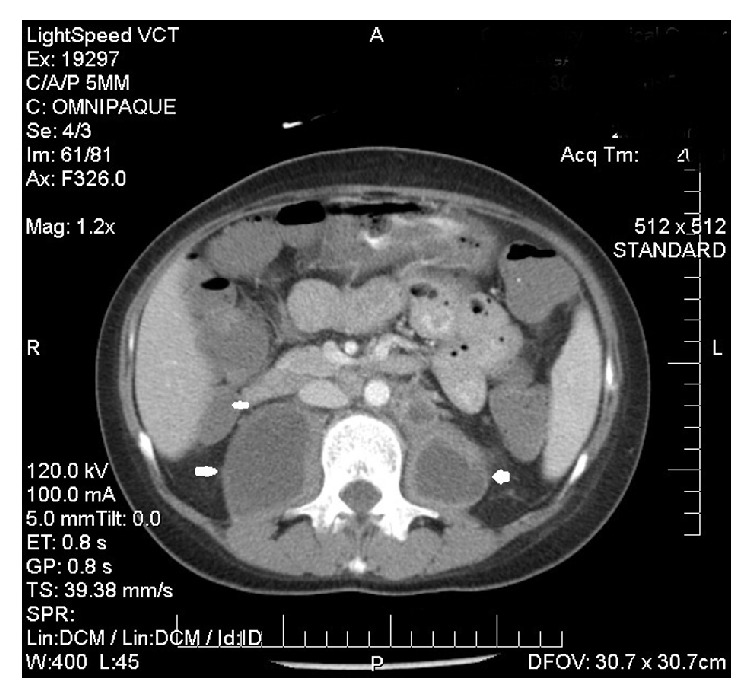
Abdominal CT scan demonstrating large hypodense iliopsoas fluid collections (white arrows).

A 32-year-old Asian woman received a successful two-antigen mismatched deceased donor renal transplant. She underwent Alemtuzumab and Methylprednisone induction. The immediate posttransplant course was uncomplicated. She was discharged with a nadir serum creatinine of 0.9 mg/dL. Immunosuppression consisted of Mycophenolic acid delayed release (MMF) 720 mg twice daily and Tacrolimus therapy (target trough 9–12 ng/mL). Three months after transplant the patient developed elevated liver enzymes associated with fever of 102°F. Initial blood cultures, CMV, parvovirus B-19 and EBV by quantitative polymerase chain reaction were negative. Computed tomographic imaging studies (CT) revealed periaortic lymphadenopathy (Figure 1). Liver biopsy revealed noncaseating granulomas and no evidence of posttransplant lymphoproliferative disorder (Figure 2(a)). Para-aortic lymph node biopsy revealed acid fast bacilli which were confirmed to be Mycobacterium tuberculosis (MTB) by nucleic acid amplification test and culture (Figure 2(b)). Review of pretransplant records revealed that the patient developed a 5 mm induration on tuberculin skin testing. Based on a history of prior administration of the BCG vaccine and a completely normal radiograph of the chest, the decision to forego Isoniazid prophylaxis against latent TB was made. Treatment was begun with Isoniazid 300 mg daily, Rifampin 450 mg daily, Ethambutol 800 mg daily, and Pyrazinamide 1000 mg daily. Her immunosuppression was reduced to maintain target Tacrolimus trough levels of 6 ng/mL and MMF was decreased to 180 mg twice daily. Resolution of symptoms occurred after initiation of therapy and reduction of immunosuppression. Five months after initiation of therapy the patient developed fever, weight loss, back pain, and weakness. CT scan demonstrated multiple lobulated peripherally enhancing fluid collections within the iliopsoas muscles as well as within the left hepatic lobe, most suggestive of abscesses (Figure 3). Empirical intravenous Ampicillin/Sulbactam was initiated at 3.1 grams every 8 hours. She underwent drainage of fluid collections, and gram stain, and cultures were obtained. Gram stain and acid fast stain of fluid revealed multiple neutrophils and no organisms. Fluid cultures revealed no growth of bacteria or MTB. The diagnosis of IRIS was established, corticosteroids were not administered, and the patient was maintained on two-drug anti-TB therapy consisting of Rifampin and Isoniazid. During the course of therapy, she was rehospitalized for fever of unknown origin.

**Figure 4:**
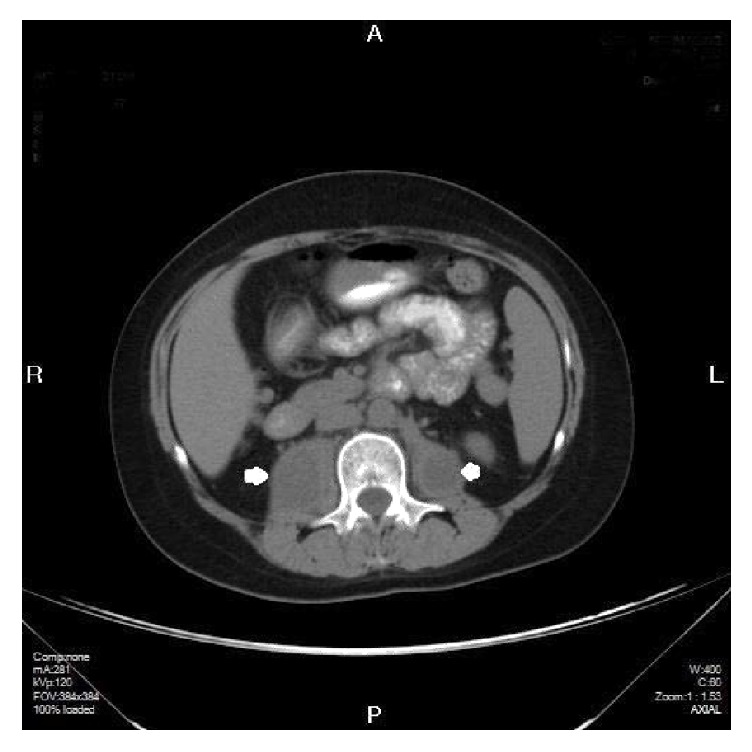
Abdominal CT scan demonstrating resolving iliopsoas fluid collections.

**Figure 5:**
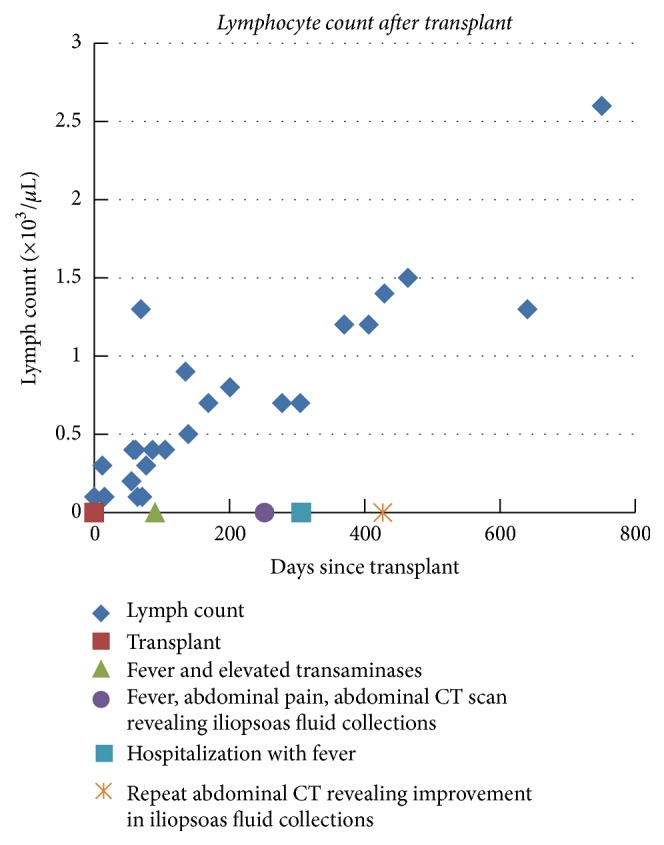
Timeline of clinical events and total lymphocyte count (diamond) from the time of transplant (red square), onset of elevated transaminases and fever (triangle), development of fever and abdominal masses (circle), rehospitalization with fever (blue square), and clinical improvement (star).

Fever, abdominal pain, and fullness slowly resolved over the course of therapy. Repeat CT imaging of the abdomen revealed resolving iliopsoas fluid collections (Figure 4). After 4 months of 4-drug regimen, the patient was continued on Rifampin and Isoniazid. The patient finished one year of antituberculosis therapy and has remained symptom-free with a functioning allograft. This patient received lymphocyte depleting induction with Alemtuzumab and her immunosuppression was reduced after the diagnosis of active TB. As IRIS may occur in the setting of treatment for TB and immune reconstitution, the time course of her illness and her total lymphocyte count are displayed (Figure 5).

## 3. Discussion

SOT recipients have a high risk of developing TB during the posttransplant period usually due to reactivation of latent infection [1, 2, 4]. MTB infection in SOT can result in extremely high mortality rates and high rates of organ rejection [1, 3, 4, 7]. Extrapulmonary and disseminated TB may occur in up to 12–50% of posttransplant TB cases and is particularly challenging as it may mimic other infectious and noninfectious complications observed in the posttransplant course such as PTLD and systemic opportunistic fungal and viral infections [3–5, 7]. Drug toxicity and drug interactions are important issues to consider in SOT recipients undergoing antimycobacterial therapy [1, 4, 6, 7].

Rifampin induces hepatic and intestinal CYP3A4 resulting in protracted difficulty in maintaining therapeutic drug levels of calcineurin inhibitors (CNI), inhibitors of the mammalian target of Rapamycin (MTOR), and corticosteroids [11, 12]. Rifampin also induces intestinal, renal, and hepatic enzymes involved in the conversion of MMF into the active mycophenolic acid (MPA) resulting in marked decrease in active drug [13]. Acute rejection may occur and IRIS may develop as immunosuppression is decreased by clinicians and as concurrent drug-drug interactions occur [1, 2, 4, 9]. Thus, in the clinical setting of transplantation, treatment of TB can result in rejection rates and allograft loss in 33% of cases [1, 2, 4, 7].

IRIS can be described as a pathological hyperinflammatory host response when reversal of either pathogen or iatrogenic immunosuppression occurs resulting in paradoxical worsening of disease during effective antimicrobial therapy [9]. IRIS has been reported commonly in the HIV/AIDS population with MTB receiving HAART [9, 10]. In SOT, IRIS has been reported in recipients undergoing treatment for mycotic infections, BK polyoma nephropathy, and systemic cytomegalovirus (CMV) [9]. IRIS has recently been described with increasing frequency in SOT recipients who are undergoing treatment for TB and other opportunistic infections who have necessarily had to have their immunosuppression curtailed [9, 10, 14]. Review of the literature documenting IRIS in SOT has revealed 3 case reports and 1 retrospective study documenting 9 cases, which include 6 liver, 4 kidney, 1 heart, and 1 heart lung transplant [10, 14–16].

In SOT undergoing treatment for active TB, IRIS usually occurs within 3 months [10]. Risk factors appear to be liver transplantation, use of Rifampin, CMV infection, and the presence of extrapulmonary TB [10]. Although IRIS has been increasingly reported in solid organ transplant recipients, this entity may still remain unrecognized.

Although a working paradigm of the pathophysiology of IRIS occurring in the posttransplant setting has not been established, current evidence suggests an imbalance between pathogen directed host inflammatory (Th1, Th17) and anti-inflammatory (Th-2, Tregs) effector cells resulting in hyperinflammatory response to a pathogen with ensuing tissue damage [9]. Both MTB infection and immunosuppressive therapy have effects on T cell repertoire resulting in polarization towards a Th2 cell response [9, 10, 17]. TB infection results in the modulation of the immune response by increasing the generation of Cd4 Th2 subtypes and Treg cells [9, 17, 18]. In the clinical setting of transplantation the polarization of Cd4 cells into Th1 and Th17 subtypes mediates allograft rejection [9]. Conversely, increases in Tregs and polarization towards Cd4 Th2 cells lead to degrees of tolerance. Calcineurin inhibitors (CNI), mycophenolate (MMF), corticosteroids, and MTOR inhibitors decrease Th1 and Th2 generation [9, 19]. Furthermore, corticosteroids increase Th2 and Treg generation [9, 19]. MTOR inhibitors result in decrease in Th1, Th2, and Th17 cells while increasing generation of Tregs [9, 19, 20]. Induction therapy with the lymphocyte depleting antibody Alemtuzumab and antithymocytic globulins results in decreases in Th1 and TH17 Tcell repertoire and preferential increases in Treg cells during the proliferation and recovery period that occurs after T cell depletion [19, 21].

It is important to also consider epidemiological data involving TB to further examine high risk population subgroups within which our patient resides. According to the Centers for Disease Control and Prevention (CDC), one-third of the world's population is infected with TB [22]. In 2015, the TB incidence rate per 100,000 persons has remained relatively stable at approximately 3.0 since 2013 [22]. Alternative data collected from 1993 to 2006 found that almost one-fifth of US tuberculosis cases are extrapulmonary in nature [23]. There is an especially high incidence of TB in the United States in Asian patients. According to a report on tuberculosis trends conducted by the CDC, compared to non-Hispanic Caucasians, the TB incidence rate among the Asian population was 28.5 times greater, at 18.2 cases per 100,000 persons, the highest among all racial groups [24]. With respect to patients with chronic renal failure on hemodialysis, there is also a high prevalence of latent TB infection. Population-based cohort studies have designated relative risks of TB ranging from 3.4 to 25.3 in dialysis patients compared with the general population [25]. The use of the anti-CD52 monoclonal antibody, Alemtuzumab, has also been linked to the development of various opportunistic infections, including TB. In a Hong Kong study, researchers recommended TB prophylaxis in those who are administered Alemtuzumab after 7 out of 27 immunosuppressed patients developed TB, 3 of which were extrapulmonary [26]. However, it must be noted that the large variety of potential infections with Alemtuzumab therapy makes it difficult to have universal prophylaxis strategies and rather requires vigilant surveillance and awareness by the treating physician [27]. This is true not only for those treated with Alemtuzumab, but for all high risk population subgroups.

## 4. Conclusion

Immune Reconstitution Inflammatory Syndrome (IRIS) has primarily been studied in the context of HIV/AIDS-associated paradoxical exacerbation of infection symptoms after recovery of an immunosuppressed state. Although there have been increasing reports of IRIS occurring in the setting of SOT and infections with MTB and a variety of opportunistic infections, we believe that IRIS is an underrecognized complication in this high risk group. This report demonstrates the difficulties encountered when IRIS develops in a SOT recipient during the management of extrapulmonary MTB infection by reduction in immunosuppression and institution of appropriate antimycobacterial therapy. It is possible that decreasing immunosuppression may have further exacerbated the development of IRIS in this patient. The potential for the development of IRIS in SOT with MTB may have an impact on treatment strategies for physicians managing transplant immunosuppression. Clinicians should therefore be cognizant of the potential of IRIS not only in HIV/AIDS cases but also in solid organ transplant patients. Caution should be used in decreasing immunosuppression in SOT patients undergoing treatment for MTB. Developing greater awareness among treatment providers about the paradoxical nature of IRIS is essential for the safe and proper management of patients that have undergone therapeutic immunosuppression for transplantation. What is clear is that further investigation into IRIS associated with solid organ transplantation must be completed to develop not only our understanding but also appropriate treatment protocol for this complex phenomenon.
